# A concept of natural genome reconstruction.Part 2. Effect of extracellular double-stranded DNA fragments on hematopoietic stem cells

**DOI:** 10.18699/vjgb-24-106

**Published:** 2024-12

**Authors:** V.S. Ruzanova, S.G. Oshikhmina, A.S. Proskurina, G.S. Ritter, S.S. Kirikovich, E.V. Levites, Y.R. Efremov, T.V. Karamysheva, M.I. Meschaninova, A.L. Mamaev, O.S. Taranov, A.S. Bogachev, S.V. Sidorov, S.D. Nikonov, O.Y. Leplina, A.A. Ostanin, E.R. Chernykh, N.A. Kolchanov, E.V. Dolgova, S.S. Bogachev

**Affiliations:** Institute of Cytology and Genetics of the Siberian Branch of the Russian Academy of Sciences, Novosibirsk, Russia; Institute of Cytology and Genetics of the Siberian Branch of the Russian Academy of Sciences, Novosibirsk, Russia Novosibirsk State University, Novosibirsk, Russia; Institute of Cytology and Genetics of the Siberian Branch of the Russian Academy of Sciences, Novosibirsk, Russia; Institute of Cytology and Genetics of the Siberian Branch of the Russian Academy of Sciences, Novosibirsk, Russia; Institute of Cytology and Genetics of the Siberian Branch of the Russian Academy of Sciences, Novosibirsk, Russia; Institute of Cytology and Genetics of the Siberian Branch of the Russian Academy of Sciences, Novosibirsk, Russia; Institute of Cytology and Genetics of the Siberian Branch of the Russian Academy of Sciences, Novosibirsk, Russia; Institute of Cytology and Genetics of the Siberian Branch of the Russian Academy of Sciences, Novosibirsk, Russia; Institute of Chemical Biology and Fundamental Medicine of the Siberian Branch of the Russian Academy of Sciences, Novosibirsk, Russia; Laboratory Angiopharm LLC, Novosibirsk, Russia; State Scientific Center of Virology and Biotechnology “Vector” of Rospotrebnadzor, Koltsovo, Novosibirsk region, Russia; Novosibirsk State University, Novosibirsk, Russia; City Clinical Hospital No. 1, Novosibirsk, Russia; Novosibirsk Tuberculosis Research Institute, Novosibirsk, Russia; Research Institute of Fundamental and Clinical Immunology, Novosibirsk, Russia; Research Institute of Fundamental and Clinical Immunology, Novosibirsk, Russia; Research Institute of Fundamental and Clinical Immunology, Novosibirsk, Russia; Institute of Cytology and Genetics of the Siberian Branch of the Russian Academy of Sciences, Novosibirsk, Russia; Institute of Cytology and Genetics of the Siberian Branch of the Russian Academy of Sciences, Novosibirsk, Russia; Institute of Cytology and Genetics of the Siberian Branch of the Russian Academy of Sciences, Novosibirsk, Russia

**Keywords:** hematopoietic stem cells, extracellular DNA, internalization, terminal differentiation, single-strand breaks, гемопоэтические стволовые клетки, экстраклеточная ДНК, интернализация, терминальная дифференцировка, одноцепочечные разрывы

## Abstract

In this part of the study, the first component of the concept of “natural genome reconstruction” is being proven. It was shown with mouse and human model organisms that CD34+ hematopoietic bone marrow progenitors take up fragments of extracellular double-stranded DNA through a natural mechanism. It is known that the process of internalization of extracellular DNA fragments involves glycocalyx structures, which include glycoproteins/protein glycans, glycosylphosphatidylinositol-anchored proteins and scavenger receptors. The bioinformatic analysis conducted indicates that the main surface marker proteins of hematopoietic stem cells belong to the indicated groups of factors and contain specific DNA binding sites, including a heparin-binding domain and clusters of positively charged amino acid residues. A direct interaction of CD34 and CD84 (SLAMF5) glycoproteins, markers of hematopoietic stem cells, with double-stranded DNA fragments was demonstrated using an electrophoretic mobility shift assay system. In cells negative for CD34, which also internalize fragments, concatemerization of the fragments delivered into the cell occurs. In this case, up to five oligonucleotide monomers containing 9 telomeric TTAGGG repeats are stitched together into one structure. Extracellular fragments delivered to hematopoietic stem cells initiate division of the original hematopoietic stem cell in such a way that one of the daughter cells becomes committed to terminal differentiation, and the second retains its low-differentiated status. After treatment of bone marrow cells with hDNAgr, the number of CD34+ cells in the colonies increases to 3 % (humans as the model organism). At the same time, treatment with hDNAgr induces proliferation of blood stem cells and their immediate descendants and stimulates colony formation (mouse, rat and humans as the model organisms). Most often, the granulocyte-macrophage lineage of hematopoiesis is activated as a result of processing extracellular double-stranded DNA. The commitment process is manifested by the appearance and repair of pangenomic single-strand breaks. The transition time in the direction of differentiation (the time it takes for pangenomic single-strand breaks to appear and to be repaired) is about 7 days. It is assumed that at the moment of initiation of pangenomic single-strand breaks, a “recombinogenic situation” ensues in the cell and molecular repair and recombination mechanisms are activated. In all experiments with individual molecules, recombinant human angiogenin was used as a comparison factor. In all other experiments, one of the experimental groups consisted of hematopoietic stem cells treated with angiogenin

## Abstract (ru)

В настоящей части исследования доказывается первая составляющая концепции «природной реконструкции генома». На модельных организмах мышь и человек показано, что CD34+ гемопоэтические предшественники костного мозга захватывают фрагменты экстраклеточной двуцепочечной ДНК естественным природным механизмом. Известно, что в процессе интернализации экстраклеточных фрагментов ДНК принимают участие структуры гликокаликса, в состав которых входят гликопротеины/протеогликаны, гликозилфосфатидилинозитол-заякоренные белки и скавенджер-рецепторы. Проведенный биоинформационный анализ свидетельствует, что основные поверхностные маркерные белки гемопоэтических стволовых клеток относятся к указанным группам факторов и содержат специфические сайты связывания ДНК, включающие гепарин-связывающий домен и кластеры положительно заряженных аминокислотных остатков. С использованием системы Electrophoretic mobility shift assay показано прямое взаимодействие CD34 и CD84 (SLAMF5) гликопротеинов, маркеров гемопоэтических стволовых клеток, с фрагментами двуцепочечной ДНК. В клетках, негативных по CD34, также интернализующих фрагменты, происходит конкатемеризация доставленных внутрь клетки фрагментов. При этом в одну структуру сшивается до пяти мономеров олигонуклеотидов, содержащих девять теломерных повторов TTAGGG. Доставленные в гемопоэтические стволовые клетки экстраклеточные фрагменты инициируют деление исходной гемопоэтической стволовой клетки таким образом, что одна из дочерних клеток уходит в терминальную дифференцировку, а вторая сохраняет свой низкодифференцированный статус. В составе колоний после обработки клеток костного мозга препаратом hDNAgr количество СD34+ клеток возрастает до 3 % (модельный организм – человек). Одновременно обработка препаратом hDNAgr индуцирует пролиферацию стволовых клеток крови и их ближайших потомков и стимулирует колониеобразование (модельные организмы – мышь, крыса, человек). Наиболее часто в результате обработки экстраклеточной двуцепочечной ДНК активируется гранулоцитарно-макрофагальный росток кроветворения. Процесс коммитирования манифестируется появлением и репарацией пангеномных одноцепочечных разрывов. Время перехода в направлении дифференцировки (время появления и репарации пангеномных одноцепочечных разрывов) составляет около 7 суток. Предполагается, что в момент инициации пангеномных одноцепочечных разрывов в клетке создается «рекомбиногенная ситуация» и активируются молекулярные репаративно-рекомбинационные механизмы. Во всех проведенных экспериментах по анализу индивидуальных молекул в качестве фактора сравнения использовался ангиогенин рекомбинантный человеческий. Во всех других экспериментах одной из сравниваемых групп являлись гемопоэтические стволовые клетки, обработанные ангиогенином.

## Introduction

Hematopoietic stem cell (HSC) and its bone marrow (BM) niche constitute a unique cell system, which maintains the balance of blood cell elements and repairs tissue and organs throughout life. The HSC concept is complex; it characterizes a number of cellular states and various cell types of different anatomical localization, developing into different cell lineages. Three HSC classes are distinguished: myeloid-biased, lymphoid-biased, and balanced cells; all of them vary in their differentiation capacity, which is fixed epigenetically. Clonal analysis indicates that these cell classes are comprised of two populations: short-lived HSCs and long-lived progenitors. The first cell population enters the differentiation and proliferation phase within a few weeks, while long-lived progenitors remain in the quiescent G0 phase for a long time (Muller-Sieburg, Sieburg, 2008).

It is generally believed that long-lived quiescent mouse HSCs have the following phenotype: Lin– Kit+ Sca-1+ CD150+ CD34– Flk2– CD48–. There are 30,000 BM mononuclear cells per one HSC, and about 80 % of HSCs remain quiescent throughout life (in humans), preserving their stemness (Morita et al., 2010; Zhang, Sadek, 2014; Wilkinson et al., 2020).

The HSC is surrounded by different cell types; these cells create a niche for the implementation of HSC functions. The stem cell niche is composed of endothelial cells, multiple mesenchymal cells (adipocytes, CXCL12+, adventitial reticular [CAR] cells, osteoclast-like cells [OLCs], leptinR+ and nestin+ cells, and NG2+ arteriolar wall cells), non-myelin- forming Schwann cells, and hematopoietic cells (macrophages and megakaryocytes) (Lévesque et al., 2010; Mendelson, Frenette, 2014; Kumar, Geiger, 2017; Szade et al., 2018; Lucas, 2019).

Two types of HSC niches are currently distinguished in the adult human BM. The osteoblastic niche is responsible for the quiescent state of early primitive progenitors that retain stemness for a long time. Once activated, HSCs differentiate into blood precursors located within a vascular niche, adjacent to sinusoid endothelial cells (Redondo et al., 2017).

The fundamental characteristic of the primitive HSC is its immanent choice: to either maintain the quiescent state and divide symmetrically into two identical HSCs or divide asymmetrically and give rise to a committed cell with further development of a certain cell lineage.

The HSC function is directly associated with the balance between quiescence and activation. A decreased ability of the HSC to exit quiescence results in insufficient blood cell reproduction. At the same time, if an unreasonably high number of cells exit quiescence and do not return to this state after activation, the HSC pool is depleted, resulting in BM function failure (Scharf et al., 2020). HSCs of a young organism are known to divide symmetrically and proliferate more often, while progenitors in adult and aging organisms are mainly quiescent (Desterke et al., 2021).

The establishment of the HSC state involves numerous factors. First of all, these are the anatomical localization of HSCs and the stem niche preserving them, and the local hypoxia level. Hypoxia is one of the key factors determining the HSC state, and the majority of quiescent and primitive HSCs are located in hypoxic BM areas with reduced blood perfusion (Forristal, Levesque, 2014; Zhang, Sadek, 2014). Factors secreted by the stem niche and HSCs, so-called membrane-associated factors (Winkler et al., 2012; Forristal, Levesque, 2014; Goncalves et al., 2016; Silberstein et al., 2016; Redondo et al., 2017; Chen T.L. et al., 2018; Scharf et al., 2020; Desterke et al., 2021), are important participants of the processes determining the HSC biological state. Furthermore, the same factor can induce quiescence in one HSC type and transition to the cycle and commitment in another type, as it was shown for angiogenin (Goncalves et al., 2016). Migrating peripheral leukocytes, histamine and TNF-α secreted by them, and other BM and peripheral blood cells induce activation of quiescent progenitors (Lucas, 2019; Pinho, Frenette, 2019). Different pharmacological agents, inflammation, starvation, environmental xenobiotics, and radiation also determine the HSC’s fate (Chen T.L. et al., 2018; Scharf et al., 2020; Kiang et al., 2021; Wang et al., 2021).

Unsymmetrical division with subsequent commitment and proliferation is the basic mechanism of replenishment of blood cell populations. This process presents a finely regulated sequence of events, involving a diverse and abundant set of inducers. As previously mentioned, terminal differentiation, proliferation, and mobilization of HSCs can be activated by such environmental factors and body physiological systems as integral stimuli forming the common response vector of the HSC and its environment (the stem niche). This process results in activation of molecular signaling cascades and gene platforms determining the fate of the HSC and its committed progenitor (Kulkarni, Kale, 2020). Inflammation is one of the initiating factors in this process. As a result of the inflammatory response, a huge variety of active molecules are released into the bloodstream and lymphatic system, including a palette of pro-inflammatory cytokines, glucocorticoids (Pierce et al., 2017), granulocyte-macrophage colony-stimulating factor (GM-CSF), etc., which are the trigger releasing the resting HSC into the cycle. In addition, a large amount of apoptotic cell DNA (self-DNA) and pathogen-associated double-stranded DNA (dsDNA) and RNA appears in the bloodstream during both sterile and pathogen-induced inflammation (Jiang, Pisetsky, 2005; Saitoh et al., 2010; Lauková et al., 2019; Korabecna et al., 2020; Kananen et al., 2023). The involvement of the inflammatory process in HSC terminal differentiation indicates that all factors released into the blood during inflammation, including fragments of extracellular self/pathogen-associated DNA, affect the decision-making of primitive progenitors in a transient, competitive or restricted manner (Seita, Weissman, 2010). The inflammation is considered to shift differentiation of hematopoietic progenitors in the myeloid direction (Kovtonyuk et al., 2016).

Our recent studies have shown that stem cells of different genesis, cancer stem cells (Ritter et al., 2022), and HSCs (Potter et al., 2024) internalize extracellular dsDNA fragments through a natural mechanism. We propose that this newly discovered feature of poorly differentiated cells, including HSCs, is a transitional intermediary element in understanding the processes that take place in hematopoietic precursors, including the exit to terminal differentiation and proliferation upon their interaction with extracellular dsDNA fragments circulating in the blood.

There is another phenomenon that is the cornerstone of the concept proposed in the first part of the study. It is the presence of single-strand breaks (nicks) in the stem cell genome and their association with terminal differentiation of progenitors.

This phenomenon was first reported in studies conducted on a series of eukaryotic models at the end of the previous century. To analyze the events occurring in the nuclear chromatin during commitment, the following inducers were used: DMSO, sodium butyrate, butyrylcholine, and retinoic acid. Single-strand breaks were detected using sedimentation assay (Jacobson et al., 1975; Scher, Friend, 1978), hydroxyapatite chromatography (Pulito et al., 1983), alkaline filter elution (McMahon et al., 1984; Boerrigter et al., 1989; Kaminskas, Li, 1989), in situ nick translation (Iseki, 1986; Patkin et al., 1995), and alkaline electrophoresis (McMahon et al., 1984; Vatolin et al., 1997). It turned out that formation and repair of single-strand breaks is a dose- and time-dependent process that does not correlate with the direction of differentiation (Scher, Friend, 1978; Farzaneh et al., 1982).

Chromatin nicking was shown to be associated with the activity of calcium/magnesium-dependent DNases, i. e. it is an enzymatic process, and single-strand breaks occur randomly (McMahon et al., 1984; Kaminskas, Li, 1989). Repair of single-strand breaks involves ADP-ribosyl transferase, which, in turn, is also believed to regulate differentiation through stimulation of ligase activity (Farzaneh et al., 1982; Johnstone, Williams, 1982). Quite peculiar and complex results were obtained in the study (Patkin et al., 1995). In this work, using in situ nick translation, the authors established that metaphase chromosomes in stem cells contain numerous nicks in the phase of transition to a committed state.

Thus, the presence of single-strand breaks was shown to closely correlate with terminal differentiation of stem cells. This event is considered the earliest manifestation of initiated commitment. These breaks are not associated with apoptosis, they do not result in cell death, and chromatin integrity is restored after a certain time. A possible explanation for this phenomenon is activation of genes necessary for commitment at this point in time (Jacobson et al., 1975; Farzaneh et al., 1982). We believe that this phenomenon is the cornerstone of the entire differentiation process: a biological, supramolecular, and large-scale manifestation of a change in the cell biological status. It is pangenomic single-strand breaks that allow the cell, apparently with minimal energy costs, to reorganize the chromatin topology of the undifferentiated state into a new architecture required for cell specialization (which, naturally, is associated with a fundamental change in the platform of expressed genes, as follows from the reasoning in the work (Jacobson et al., 1975; Farzaneh et al., 1982)). This is the phenomenon we attempted to characterize in the current part of the study within the new experimental framework, where extracellular dsDNA fragments act as the inducer.

Unfortunately, we did not manage to find studies on the presence and repair of single-strand breaks in hematopoietic stem cells in the available literature for the past 20 years. It is absolutely unclear why this area characterizing terminal transition of poorly differentiated stem cells of various origin has not received further development.

Therefore, in the second part of the work cycle, we analyzed internalization of dsDNA fragments in cells and their induction of terminal differentiation of progenitors, which manifests itself in the formation and repair of pangenomic single-strand breaks.

## Materials and methods

Experimental animals. The following animals were used in the study: male CBA/Lac mice aged 2–5 months, 9–12 months old male CBA/Lac mice, male Wistar rats aged 2–6 months, and 18–22 months old male Wistar rats. All animals were bred at the Conventional Vivarium of the Institute of Cytology and Genetics of the Siberian Branch of the Russian Academy of Sciences (Novosibirsk, Russia). Animals were kept in groups of 6–10 mice and 3–4 rats per cage with free access to food and water. All animal experiments were approved by the Animal Care and Use Committee of the Institute of Cytology and Genetics of the Siberian Branch of the Russian Academy of Sciences. Mice were withdrawn from the experiment by cervical dislocation, and rats were either euthanized using CO2 or decapitated.

Human bone marrow cells. Cryopreserved bone marrow cells from patients with Hodgkin lymphoma were used in the study. Cells were provided by the Cryobank of the Research Institute of Fundamental and Clinical Immunology.

hDNAgr. The hDNAgr preparation (DNA genome reconstructor) was isolated from placentas of healthy women. Total genome DNA was fragmented to 1–20 nucleosome monomers (200–2,000 bp) by ultrasonic disintegration, deproteinized using proteinase K, and extracted with phenol- chloroform.

Angiogenin. Recombinant human angiogenin was provided by Angiopharm Laboratory LLC (Novosibirsk, Russia). Angiogenin was labeled with Cy5 according to the manufacturer’s instructions (Lumiprobe, Germany).

TAMRA-labeled DNA probe. Human AluI repeat DNA was labeled with the fluorescent dye TAMRA by PCR using TAMRA-5′-dUTP (deoxyuridine triphosphate) as described in (Dolgova et al., 2014).

**Fig. 1.**
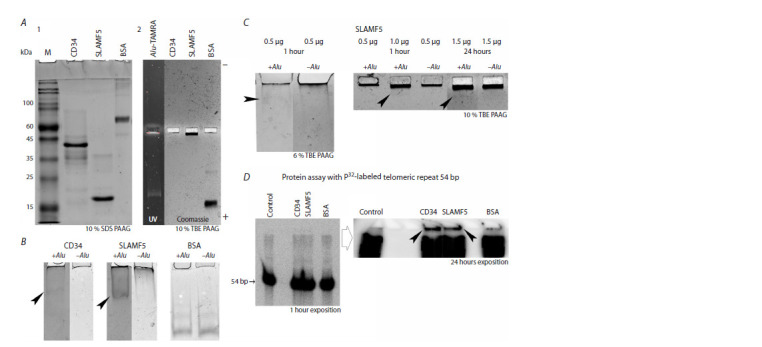
Analysis of direct molecular interaction between Alu-TAMRA/telomeric repeat dsDNA (n = 9) and HSC marker proteins CD34 and CD84 (SLAMF5). A – electrophoresis of analyzed factors in 10 % SDS (1) and 10 % native tris-borate horizontal (2) polyacrylamide gel. HSC markers do not have electrophoretic mobility in native conditions and thus do not enter the gel. The part of the gel with a dark field on the right panel demonstrates migration of the AluI dsDNA probe. B – change in electrophoretic mobility of factor samples after formation of complexes with the TAMRA-labeled AluI DNA probe. The migrating fraction of proteins (CD34 and SLAMF5) is clearly seen, which indicates that protein molecules are charged; the charge is apparently due to the DNA molecule the protein has formed a physical bond with (indicated with arrows). No changes in protein migration are detected in BSA. C – evaluation of some parameters of TAMRA AluI DNA probe-SLAMF5 complex formation. The left panel presents an electropherogram of the DNA probe-SLAMF5 complex in a native 6 % polyacrylamide gel. The amount of protein loaded on the gel is the same in control and experimental samples. The formation of a migrating protein fraction and a simultaneous decrease in its amount at the start are clearly visible. The right panel (10 % native tris-borate gel) shows the results for several modes of the DNASLAMF5 complex formation (indicated with arrows). It was found that the protein and DNA binding is not determined by time and the factor molar ratio. This fact indicates the absence of a stoichiometry between the TAMRA AluI DNA probe and SLAMF5. D – DNA-protein interactions between CD34, SLAMF5, and BSA using P32-labeled double-stranded oligonucleotide containing 9 telomeric repeats (54 bp). Specific interactions between DNA and proteins are clearly detected in the CD34 and SLAMF5 samples (indicated with arrows).

Assessment of change in gel mobility of the complex of CD34 and SLAMF5 proteins and DNA probes. To analyze the interaction of the CD34 and SLAMF5 proteins with TAMRA-labeled DNA probe and P32-labeled double-stranded (TTAGGG)9 telomeric repeat, protein and DNA samples were incubated at different ratios and for different time periods in 10 mM PBS buffer at 37 °C (see Figure 1 caption). Incorporation of γP32-ATP and native polyacrylamide gel electrophoresis were performed according to standard procedures (Maniatis et al., 1984; DNA Cloning…, 1985).

Isolation of bone marrow cells. To isolate the BM, animals were withdrawn from the experiment, femurs and tibias were isolated, epiphyses were removed, and BM cavity was washed with IMDM + 2 % FBS. The resulting cell suspension was passed through a 21-gauge needle several times to eliminate BM rosettes and then through a 40-μm filter. Cells were pelleted for 10 minutes at 400 g and resuspended in red blood cell lysis buffer containing 130 mM ammonium chloride for 3–5 min. The buffer was then diluted 10-fold with PBS, cells were re-pelleted, resuspended in IMDM medium, and counted in a Goryaev chamber

Internalization of DNA and angiogenin by human and mouse HSCs. To stain HSC colonies, mouse anti-Sca-1 and anti-c-Kit antibodies and 0.1 μg of TAMRA-labeled DNA were added to cells in 100 μl of IMDM medium using the manufacturer’s protocol. The resulting mixture was carefully plated in 35-mm Petri dishes with HSC colonies by avoiding the contact with methylcellulose and colonies and then spread over a small surface area. A laser scanning confocal microscope LSM 780 NLO (Zeiss) and ZenLight software were used for data collection and imaging.

To quantify TAMRA-positive (TAMRA+) cells in BM cells and colony cell suspension, 1 × 106 cells were incubated in 400 μl of IMDM supplemented with 0.1 μg of TAMRA-labeled DNA for 30 min at room temperature in the dark. Cells were pelleted for 5 min at 400 g and 25 °C, washed in a small medium volume, and resuspended in the final medium volume. The same protocol was used for staining and analysis of c-Kit+/Sca-1+/TAMRA+ cells.

For fluorescence confocal microscopy analysis, 5 μg of Cy5-labeled angiogenin with and without antibodies was added to 3 × 106 BM cells and colonies resuspended in 1 ml of cell culture medium in a 12-well plate. After 30–60-min incubation, cells were analyzed on a laser scanning confocal microscope LSM 780 NLO (Zeiss) using ZenLight software. FACS analysis of cells was performed on a BD FACSAria III flow cytometer at the Flow Cytometry Center for Collective Use of the Institute of Cytology and Genetics of the Siberian Branch of the Russian Academy of Sciences.

DNA quantification in HSCs. For incubation of HSC colony cells with the human Alu repeat, colonies obtained after BM cell induction with hDNAgr were collected from two 35-mm Petri dishes on day 10 by adding 8 ml of IMDM. Cells were pelleted by centrifugation at 400 g for 8 min, washed with 2 ml of the medium, and re-pelleted. A fragment of the human Alu repeat was added to cells to a concentration of 0.23 μg per 1 × 106 cells; the mixture was incubated for 30 min. Cells were washed, pelleted by centrifugation at 400 g for 5 min, and resuspended in 1 ml of PBS.

Real-time PCR was conducted using the BioMaster RT-qPCR kit (SYBR Green dye) (#RM03-200, Biolabmix, Russia). Standard M13 primers (M13 forward: 5′-GTAAAACGAC- GGCCA-G-3′, M13 reverse: 5′-CAGGA-AAC AG-CTATG-AC-3′) and different amounts of Alu repeat DNA (0–5,000 pg) were used to obtain the calibration curve. Each concentration was used in triplicate. The linear dependence of Ct on Alu DNA load was constructed using Bio-Rad CFX Manager v3.1 software.

Treatment of BM cells with inducers. BM cells isolated from old animals and BM sections from patients with Hodgkin lymphoma were incubated with inducers (hDNAgr or angiogenin or two inducers simultaneously) for one hour in the 5 % CO2 atmosphere with 95 % humidity at 37 °C at the following ratio: 500 μg of hDNAgr or 500 ng of angiogenin or 500 μg of hDNAgr and 500 ng of angiogenin in 1 ml in serum-free MDM medium per 3 × 106 cells. Control (untreated) BM cells were incubated in serum-free IMDM complimented with the PBS volume equal to that of the inducer added to activate BM cells. We use the term “inducer”, which designates both DNA and angiogenin in the current study, to characterize any intended and expected HSC response induced by exposure to them.

Cultivation of BM cells in methylcellulose medium. BM cells with/without inducer activation were pelleted for 10 min at 400 g and resuspended in IMDM + 2 % FBS. To quantify and analyze myeloid precursors, we placed mouse BM cells in the MethoCult M3434 methylcellulose medium, and rat and human bone marrow cells, in the MethoCult H4034 methylcellulose medium (Stem Cell Technologies). Methylcellulose analysis, colony counting, and cell isolation from methylcellulose after cultivation were carried out according to the manufacturer’s instructions. The analysis was performed in 35-mm Petri dishes, which were stored in a Petri dish of a larger diameter with additional humidification of the internal atmosphere during colony formation.

Comet tail assay for analysis of single- and doublestrand breaks. BM cells isolated from old mice and BM sections from patients with Hodgkin lymphoma after incubation in the presence/absence of inducers (hDNAgr, angiogenin, and hDNAgr+angiogenin) were cultured for 10–12 days in methylcellulose medium. Colonies isolated from methylcellulose were pooled and washed from the medium according to the manufacturer’s instructions. The resulting colony cells were counted in a Goryaev chamber and incubated with inducers. Cells were re-pelleted for 10 min at 400 g, resuspended in IMDM + 2 % FBS, placed in methylcellulose, and seeded into 24-well plates. A cell sample was collected every day at the same time (24, 48, 72, 96, 120, and 144 hrs after the start of treatment with inducers) and washed from methylcellulose. Colony cells were embedded into slow-melting 1 % agarose blocks in the amount of 5 × 103 cells per 1 block. Blocks were stored in 0.5 M EDTA at 4 °C prior to analysis. The zero point presents colony cells prior to repeated treatment with inducers.

Prior to electrophoresis, blocks were rinsed in TE buffer, incubated with a lysis buffer (50 mM EDTA, 1 % sarcosyl (Serva, Heidelberg, Germany), and 1 mg/ml proteinase K (Thermo Fisher Scientific, Waltham, USA)) for 20 min at 50 °C.

Prior to native electrophoresis, blocks were stained for 10 min in TAE buffer containing 0.5 μg/ml ethidium bromide (Medigen, Novosibirsk, Russia). Blocks were fixed on an agarose support, native electrophoresis was performed in 1×TAE buffer at 36 V and 299 mA (Model H4 Horizontal Gel Electrophoresis System (BRL, USA)) for 30 min.

Alkaline electrophoresis was carried out in a buffer containing 300 mM NaOH and 1 mM EDTA (pH > 13). Prior to alkaline electrophoresis, blocks were rinsed in the electrophoretic buffer and fixed on an agarose support. The support with blocks was placed in the electrophoretic buffer for 30 minutes. Alkaline electrophoresis was performed at 36 V and 299 mA (Model H4 Horizontal Gel Electrophoresis System chamber (BRL, USA)) for 30 min. After electrophoresis, the support with blocks was transferred to a neutral buffer containing 0.4 M Tris (pH 7.5) for 15 min. The neutral buffer was then replaced with a new one, 1 μg/ ml of ethidium bromide was added, and the support with blocks was stained for 30 min.

The support with blocks was rinsed with distilled water. Preparations were obtained and dried at 37 °C for 24 hrs. After drying, preparations were washed in distilled water for 0.5–1 h. Microscopic analysis was performed on a Zeiss Axio Imager M2 (Carl Zeiss Microscopy, Oberkochen, Germany) at the Center for Collective Use for Microscopic Analysis of Biological Objects of the Institute of Cytology and Genetics of the Siberian Branch of the Russian Academy of Sciences. Comet tail values were assessed using CASP (CASP, Wrocław, Poland) and ImageJ software.

Statistical analysis. Statistical analysis was performed using Statistica 8 software (StatSoft, USA). The reliability of differences was assessed using the Mann–Whitney U- test. Statistical significance is indicated in figure legends ( p < 0.05 or p < 0.01).

## Results

HSC capability to internalize dsDNA fragments

Our recent studies (Dolgova et al., 2014; Petrova et al., 2022; Ritter et al., 2022) report a new general biological property of stem cells of various genesis. We confirmed experimentally that mouse HSCs, as well as all poorly differentiated cells of higher eukaryotes analyzed by us, including cancer stem cells, can capture dsDNA fragments from the environment through a natural mechanism. The interaction of extracellular DNA molecules with the cell and their internalization are mediated by the glycocalyx elements of glycoproteins/proteoglycans, glycosylphosphatidylinositol- anchored proteins, and the scavenger receptor system through the caveolae/clathrin-dependent mechanism. The most important and characteristic feature is the uniqueness of the pattern of glycoproteins/proteoglycans, glycosylphosphatidylinositol-anchored proteins, and scavenger receptors located on the surface of an individual cell type. This uniqueness is determined and limited by three functional domains composed of their different representatives, namely, molecules of glycoproteins/proteoglycans, glycosylphosphatidylinositol-anchored proteins, and scavenger receptors. In other words, each stem cell can have at least three functional domains that determine its interaction with extracellular double-stranded nucleic acids and internalization of the latter. For dsDNA molecules, the heparin-binding domain, which is presented in various cell surface proteins either by the C1q domain, heparin-binding domain or the domain of positively charged amino acids, is the main binding site (Petrova et al., 2022; Ritter et al., 2022).

Is this work, we also carried out FACS and immunofluorescence analysis of the capability of human HSCs to internalize extracellular dsDNA fragments in comparison with mouse HSCs. Recombinant human angiogenin was used as a reference factor, since its effect on the cell is well-studied. We also quantified extracellular dsDNA internalized in human CD34+ HSCs.

As mentioned above, glycocalyx factors (glycoproteins/ proteoglycans, glycosylphosphatidylinositol-anchored proteins, and scavenger receptors) play a major role in DNA internalization into stem cells. We analyzed the recent literature, presenting an atlas of human HSC surface markers, for the presence of these types of proteins (Rix et al., 2022). We found that specific domains determining internalization of extracellular dsDNA fragments (clusters of positively charged amino acid residues and the heparinbinding domain) are located in the sequences of the selected proteins. The analysis results are presented in the Table. We found that several surface glycoproteins characteristic of HSCs, mainly CD34, contain domains required for internalization.

**Table 1.**
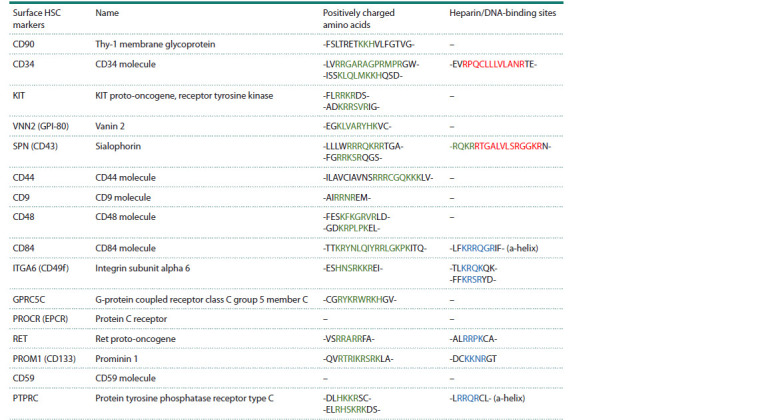
Specific human HSC surface proteins containing domains of positively charged amino acids and the heparin-binding domain Note. Clusters of positively charged amino acids are highlighted in green, DNA-binding sites are indicated in red, and heparin-binding sites are denoted in blue.

Characterization of direct molecular interaction between dsDNA Alu-TAMRA/telomeric repeat (n = 9) and HSC marker proteins CD34 and CD84 (SLAMF5). In our studies (Petrova et al., 2022; Ritter et al., 2022), we propose and confirm the hypothesis that dsDNA internalization in various stem cells is mediated by the developed glycocalyx structure on these cell membranes. The glycocalyx is composed of proteinglycans-glycoproteins, glycosylphosphatidylinositol-anchored proteins, and scavenger receptors. The interaction with these proteins is considered to have a complex physical and molecular hierar- chy, and the physical contact between dsDNA and the above factors is believed to be the basis for “dragging” dsDNA into the cell.

In the current series of experiments, we attempted to assess the possibility of a direct physical interaction between the two types of molecules: dsDNA and HSC marker proteins. The following repeats were used as the dsDNA substrate: TAMRA-labeled AluI probe, which is commonly used in the laboratory, and a telometic repeat (n = 9) in the form of P32-labeled 54-bp double-stranded oligonucleotide. CD34 and CD84 (SLAMF5) were selected as response factors. The main characteristics of the interaction between these proteins and dsDNA are presented in the Table. Experimental results are shown in Figure 1 and described in detail in the figure caption. In this part of the study, in a direct experiment, we first demonstrated the possibility of the chemical/molecular/physical interaction between dsDNA and specific HSC surface markers CD34 and SLAMF5.

Demonstration of internalization of extracellular dsDNA fragments in HSCs (Sca1+ for mouse and CD34+ for human). Using fluorescence microscopy and FACS, we demonstrated the presence of labeled dsDNA probe in human CD34+ BM cells and mouse Sca1 BM cells. Mouse primitive Sca1 hematopoietic cells and human CD34+ stem cells also internalize the reference factor human recombinant angiogenin (Supplementary Material 1)1. Analysis of the amount of dsDNA probe delivered into human CD34+ HSCs indicates that ~0.02 % of extracellular fragments (in terms of the haploid genome) are found in the internal space of this cell type. The calculations obtained are in agreement with our numerous estimates, indicating that stem cells of various genesis, depending on their origin and state, capture ~0.01–3.0 % of extracellular dsDNA fragments (in terms of the haploid genome) (Dolgova et al., 2013, 2016, 2019; Potter et al., 2018, 2024).


Supplementary Materials are available in the online version of the paper: https://vavilov.elpub.ru/jour/manager/files/Suppl_Ruzanova_Engl_28_8.pdf


**Fig.2.**
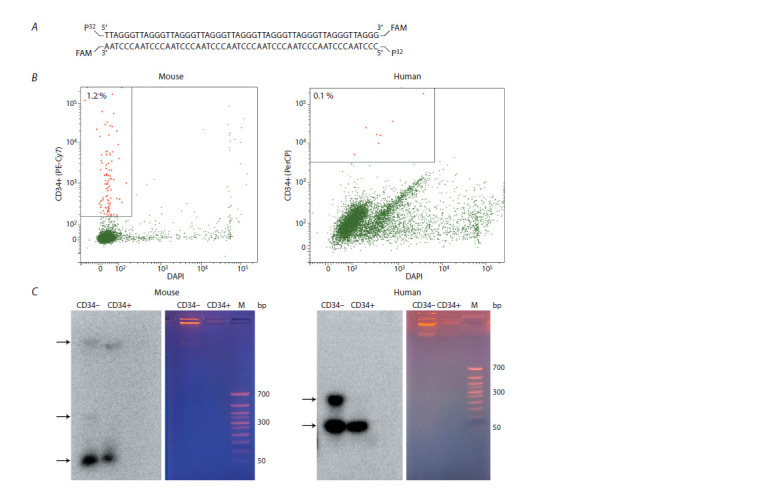
Direct experiment on internalization of extracellular dsDNA fragments. A – DNA probe structure; B – FACS analysis of mouse and human BM cell samples using the CD34 marker; C – Gel electrophoresis and autoradiography of DNA found in the internal compartments of sorted mouse and human HSCs. Arrows indicate bands corresponding to the DNA probe, concatemeric (circle?) form, and genomic DNA label.

We carried out a series of experiments that directly demonstrated internalization of extracellular DNA fragments in HSCs (Sca1+ for mouse and CD34+ for human) derived from BM cells (Fig. 2A, B). Molecule internalization in the cell includes the following phases: mobilization on the cytoplasmic membrane, internalization, and the presence and processing stage. In this regard, in order to avoid speculations on whether DNA molecules mobilized on the cytoplasmic membrane are detected in the experiment, we developed and applied a protocol of cell sample preparation, which is described in Supplementary Material 2.

It can be seen that original dsDNA probe molecules developed into forms containing up to 6–7 repeats (300– 350 bp) of the original fragment (54 bp) (indicated with black arrows) in cells negative for both mouse and human HSC markers (Fig. 2C). This fact is in good agreement with our previous results (Dolgova et al., 2013; Potter et al., 2018, 2024). In addition, the presence of labeled material in the genomic DNA fraction is clearly noted in the mouse model.

The present study was not intended to provide a deep analysis of cell populations capable of capturing extracellular DNA. This study is focused exceptionally on internalization. Similar to our previous works, the study results show that CD34+ cells capture extracellular DNA. In addition, we also showed that a population of CD34– cells, which is also present in the BM, is capable of internalizing extracellular dsDNA fragments; this population may include any variants of both multipotent progenitors and committed progeny.

Terminal differentiation, HSC proliferation, and formation of colonies induced by angiogenin, hDNAgr, and (angiogenin+hDNAgr)

Deproteinized human genomic dsDNA fragmented to 1–10 nucleosome monomers, namely hDNAgr, or genome reconstructor, was used in the study. The length of 1–10 nucleosome monomers is the physiological size of DNA molecules (self-DNA) in apoptotic cells, which are always present in the peripheral blood. The inducer human recombinant angiogenin was used as a comparison factor.

We performed a series of experiments on analysis of the stimulation of colony formation and proliferative activity of BM HSCs after treatment with the selected inducers in three models: mouse BM cells, rat BM cells, and cryopreserved human BM cells. We found that cell treatment with angiogenin, hDNAgr, and angiogenin+hDNAgr stimulates colony formation (an increase in the total number) in the studied models. The number of new colonies in mouse and human models in some cases increased by 20–30 % when using hDNAgr (Supplementary Material 3, Fig. 1A, C). A significant increase in the number of colonies was noted in the mouse model after treatment with both angiogenin and angiogenin+hDNAgr.

Angiogenin reliably stimulates cell proliferation in growing colonies in the mouse model. CFU–GM is the main responsive lineage, which is reliably confirmed in the human model. Treatment of BM cells with activators neither induces apoptosis nor stimulates CD34+ cell survival. Addition of hDNAgr and angiogenin+hDNAgr to freshly thawed human samples enhances CD34+ cell proliferation. At the same time, angiogenin neither shows any stimulatory effect nor affects the ability of hDNAgr to enhance CD34+ cell proliferation (Supplementary Material 3, Fig. 1).

A comparison was also made of the proliferative activity of CD34+ HSCs for the synthesis of the proliferative factor Ki-67 after treatment with inducers before seeding on methylcellulose and the proliferative activity of these cells, expressed in the number of cells per colony after incubation on methylcellulose for 11–15 days. No correlation was found between the two parameters (Supplementary Material 3, Fig. 2).

Assessment of the ability of colony cells selected on days 7 and 15 of culturing in methylcellulose to internalize a TAMRA-labeled 500 bp PCR fragment

The main keynote of all our studies is the confirmed statement that extracellular DNA fragments are captured by primitive stem progenitors. In humans, these cells are CD34+ progenitors. In the study performed in a mouse model (Potter et al., 2024), we showed that the number of primitive hematopoietic progenitors increases significantly in colonies formed after induction of terminal differentiation by extracellular dsDNA fragments. This makes it possible to use these progenitors to analyze various events occurring in HSCs, which is impossible in case of BM HSCs.

A similar study was conducted in a human cryopreserved BM cell model. We estimated the percentage of CD34+ stem cells in colonies formed by HSCs after their induction in BM by angiogenin, hDNAgr, and angiogenin+hDNAgr. Treatment of HSCs in BM by hDNAgr on day 15 of culturing resulted in an increase in the number of cells in the colony to 2.7 % versus 1.56 % in an individual experiment (GM-CSF-stimulated BM cells). This indicates that colonies contain a sufficient number of cells able to internalize extracellular genetic material in an amount required for reliable detection of extracellular DNA in the cell. At the same time, neither angiogenin nor angiogenin+hDNAgr increased the number of hematopoietic precursors in colonies (Supplementary Material 4).

Analysis of formation of pangenomic single-strand breaks in the cells of colonies of primitive progenitor descendants treated by hDNAgr as part of BM cells

Early studies analyzed in the Introduction section showed that the genome of embryonic stem cells is exposed by pangenomic single-strand breaks during commitment upon induction of terminal differentiation. These single-strand breaks are repaired without causing cell death. We believe that this process is important for the change in chromatin architecture characterizing undifferentiated blood stem cells to the spatial organization of expressing genes characteristic of committed progeny (Jacobson et al., 1975; Scher, Friend, 1978; Farzaneh et al., 1982; McMahon et al., 1984; Boerrigter et al., 1989; Kaminskas, Li, 1989; Vatolin et al., 1997).

We hypothesized that this process is common for all types of primitive progenitors, including HSCs. The analysis performed in the first part of our study and in the work (Potter et al., 2024) demonstrated that the selected in- ducers cause colony formation and terminal differentiation of activated BM HSCs in mice, rats, and humans. This means that formation of pangenomic single-strand breaks may also be an integral part of HSC biology. The content of HSC colonies in mice was 12–15 % (Potter et al., 2024). In human, the cell content is ~3 % (Supplementary Material 4). This indicates that there will be a sufficient number of cells retaining the undifferentiated state and undergoing terminal differentiation in the colony formed by BM HSCs after a single induction of BM cells and repeated induction of colony cells on day 15 for identification of single-strand breaks.

The work was performed in mouse and human models using the following inducers: hDNAgr, angiogenin, and angiogenin+hDNAgr. We also quantified single-strand breaks in the DNA of colony cells on day 15 after all the procedures described above.

**Fig. 3.**
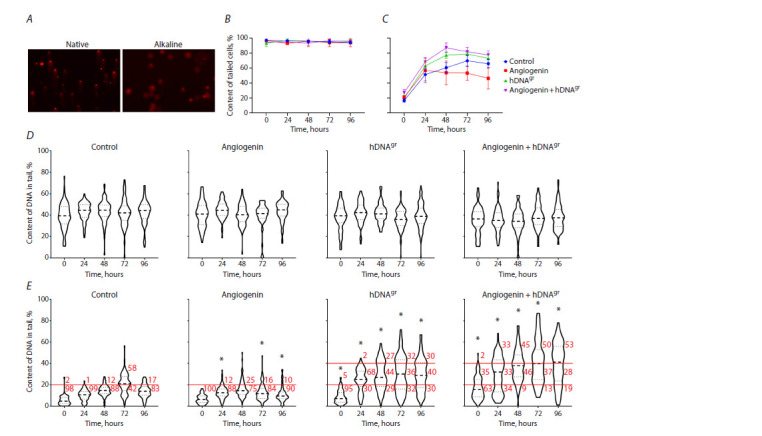
Human model. A – cells and comet tails in native and alkaline electrophoresis. B, C – content of cells with a tail in native (B) and alkaline (C) electrophoresis. D, E – diagrams showing the number of cells with different tail DNA levels in native (D) and alkaline (E) electrophoresis. The bold dashed line indicates the median value, the thin dashed line shows the interquartile range. The percentage of cells with the tail DNA level of 0–20 %, 20–40 % and >40 % is indicated in red (the corresponding ranges are highlighted with red lines). * Significant differences compared to the control group, p <0.01, Mann–Whitney test.

The analysis revealed significant and reliable differences in the studied parameters between different sample and control points (Fig. 3). An increase in the number of cells with the maximum level of tail DNA after 72–96 hrs and 96 hrs of incubation of hDNAgr-treated cells was noted in the human and mouse models, respectively. The use of angiogenin alone has virtually no effect on the induction of single-strand breaks and increase in the tail DNA content. Apparently, complete repair of single-strand breaks takes place on days 7–9 of incubation in the human model (Supplementary Material 5).

**Fig. 4.**
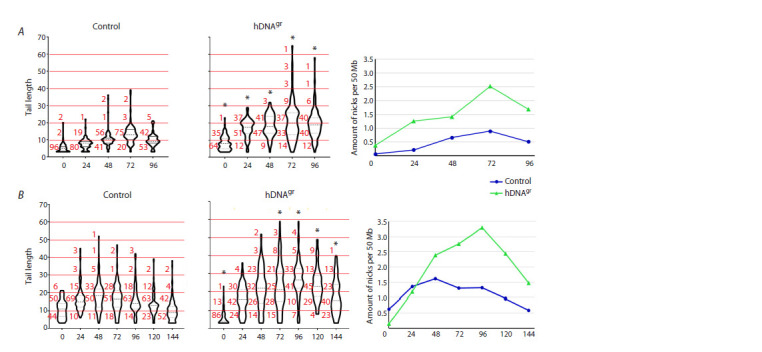
Results of two independent experiments in the human model (A, B). Diagrams for control and hDNAgr-treated cells are presented; they show the comet tail length in arbitrary units (Y axis) and time intervals with a 24-h step (X axis). The percentage of cells with the comet tail length within the corresponding interval is shown in red. * Reliable differences compared to the control group, p < 0.01, Mann–Whitney test. Graphs on the right show dependence of the calculated number of nicks per 50 × 106 bp (Y axis) on the time interval (X axis).

The obtained results on changes in comet tail lengths at specific time points made it possible to estimate the approximate number of induced pangenomic single-strand breaks (Fig. 4).

Several assumptions were made to estimate the number of single-strand breaks. One DNA strand of a chromosome was considered to break as a nick by forming two equal parts. Any other scenario required the use of a powerful mathematical framework, which did not correspond to the study goals. The smallest chromosome size is ~50 × 106 bp. In this regard, we calculated the number of breaks based on this length. This simplest scenario suggested that, if the DNA strand breaks into two equal parts forming a nick (alkaline conditions), then the length of the tail formed by one strand decreases by half. In case there are two nicks, each of the previous parts decreases by another half, etc. That is, if the tail length is considered 10 in scale units at the first point, it corresponds to either the absence of breaks or their native number. In that case, the tail length twice as long (20) corresponds to the formation of one break per the initial molecule length (chromosome). Thus, transfer to the next interval requires all DNA fragments formed at the previous stage to have another break. Hence, the number of breaks is estimated using the formula 2n + 1, where n is the number of breaks for the previous interval. The box thickness on the graph shows the number of cells in the specific interval. The number of breaks calculated for the interval was multiplied by the number of cells in the same interval. The average number of breaks per cell was calculated for the specified time point. Based on these data, a graph of the change in the number of breaks depending on time was constructed.

The conducted analysis demonstrated that, using the above calculation protocol, the maximum number of single-strand breaks is ~2.5–3.5 nicks per 5 × 106 chromatin bp and takes place at the time point of 72–96 hrs (for two independent experiments). The number of nicks in the control sample is in the range of 1.0–1.5 nicks per 5 × 106 chromatin bp (Fig. 4).

In a sample treated with angiogenin, a slightly higher number of nicks compared to the control sample can be detected in cells at the time point of the maximum chromatin perturbation. This does not contradict the results on colony stimulation, which demonstrate a positive effect of angiogenin on the formation of several types of colonies.

## Discussion

The discovered fact of dsDNA fragment internalization in HSCs with subsequent induction of terminal differentiation and colony formation suggested that, similar to embryonic stem cells (Vatolin et al., 1997), single-strand breaks are also induced in hematopoietic stem cells at the state of terminal differentiation. The analysis performed in the two selected models indicated a similar biological phenomenon in HSCs. Pangenomic single-strand breaks are formed, developed, and repaired in HSCs at the phase of terminal differentiation. Together with the experimental data presented in the literature, the obtained result indicates that this is a general biological process. Pangenomic singlestrand breaks are a necessary condition for reorientation of the activity of gene platforms determining the undifferentiated state to gene platforms characteristic of the committed cell state

For the past two decades, the main attention of researchers was focused on double-strand breaks and the variety of processes associated with their formation, as well as repair and recombination events mediated by these breaks in cells (So et al., 2017). Nevertheless, the scientific community has renewed its interest in nicks, or single-strand chromosome breaks, in the past years, as shown in some reviews (Xu, 2015; Vriend, Krawczyk, 2017; Maizels, Davis, 2018; Zilio, Ulrich, 2021). The keynote of the new surge of interest in nicked chromatin DNA is the forgotten concept of nick-initiated homologous recombination. The performed analysis indicates that nicks are no less important as intermediates of chromatin DNA metabolism, inducing repair and recombination processes in the cell, than double-strand breaks. However, unlike double-strand breaks, repair of single-strand breaks (nicks) much less frequently leads to fatal changes in the genome structure. Homologous recombination is the main mechanism of single-strand break repair.

The above indicates that single-strand breaks are inducers of recombinogenic state of the cell. The idea of the recombinogenic state is most fully described in our pioneering review (Likhacheva et al., 2008). The term “recombinogenic state” characterizes the activity of the cell molecular machine launched by a change in the higherorder chromatin architecture. Single-strand breaks are one of the inducers of such a change.

The main thesis in the review is that, if there are internalized extracellular dsDNA fragments in the cell in the activated recombinogenic state, these fragments become natural participants in the repair-recombination process activated by molecular mechanisms. This means that these fragments can participate in the recombination process as a natural recombination substrate. Hence, a general biological mechanism explaining the presence of extrachromosomal genetic information in the recipient genome as a result of either direct homologous integration of extracellular dsDNA fragments or formation of stable, genetically active extrachromosomal complexes has been found.

We characterized two phenomena with the involvement of dsDNA fragments in the repair-recombination process in our studies. In the work (Likhacheva et al., 2007), we demonstrated the participation of exogenous human DNA in the rescue of mouse BM progenitors from a lethal dose of gamma radiation, resulting in the survival of experimental animals. The mechanism of HSC rescue is associated with internalization of dsDNA fragments into the blood stem cell and repair-recombination correction of double-strand chromatin breaks induced by severe irradiation. In a series of other studies, we showed the involvement of extracellular dsDNA in suppressing the repair of interstrand crosslinks in tumor stem cells. The outcome of this participation is inability of the tumor stem cell to complete the repair of cytostatic-induced chromatin damage resulting in its further apoptotic death (Ruzanova et al., 2022). Numerous other studies indicate that single-strand breaks induce homologous recombination of the genetic material in the cell nucleus (Vriend, Krawczyk, 2017; Maizels, Davis, 2018).

## Conclusion

Thus, extracellular dsDNA fragments are internalized in HSCs through a natural mechanism, induce terminal differentiation of blood stem cells, and stimulate colony formation. Pangenomic single-strand breaks are the molecular manifestation of these processes. The formation of pangenomic single-strand breaks induces the recombinogenic state of the blood stem cell. During this process, extracellular dsDNA fragments can integrate into the recipient HSC genome. From a theoretical standpoint, a series of in- tegration scenarios are possible: the ends-in/ends-out mechanism, reciprocal homologous recombination, gene conversion or single-strand annealing, and non-homologous integration (Rubnitz, Subramani, 1984; Hastings et al., 1993; Li et al., 2001; Langston, Symington, 2004; Chen J.M. et al., 2007; Rass et al., 2012).

In the following parts of our research series, we present experimental evidence of both integration of extracellular dsDNA fragments into the HSC genome and formation of circular structures complexing with chromosomal DNA preserved under sever fractionation conditions. Comments on events associated with HSC terminal differentiation after extracellular dsDNA internalization are presented in Supplementary Material 6. In addition, an apparent discrepancy with the flow cytometry data, indicating that CD34+ HSCs do not disappear but, on the contrary, increase their number in colonies compared to the original BM cell sample, is discussed (Supplementary Material 4).

## Conflict of interest

The authors declare no conflict of interest.
